# Electronic health record use factors linked to efficiency and productivity: an explainable machine learning analysis

**DOI:** 10.1093/jamiaopen/ooag018

**Published:** 2026-02-25

**Authors:** Huan Li, Varada V Khanna, Nate Apathy, A Jay Holmgren, Andrew J Loza, Edward R Melnick

**Affiliations:** Department of Emergency Medicine, Yale University School of Medicine, New Haven, CT, 06519, United States; Computational Biology and Biomedical Informatics, Yale Graduate School of Arts and Sciences, New Haven, CT, 06510, United States; Biomedical Informatics and Data Science, Yale University School of Medicine, New Haven, CT, 06510, United States; Computational Biology and Biomedical Informatics, Yale Graduate School of Arts and Sciences, New Haven, CT, 06510, United States; Department of Biostatistics, Yale School of Public Health, New Haven, CT, 06510, United States; Health Policy & Management, University of Maryland School of Public Health, College Park, MD, 20742, United States; Division of Clinical Informatics and Digital Transformation, University of California, San Francisco, CA, 94143, United States; Biomedical Informatics and Data Science, Yale University School of Medicine, New Haven, CT, 06510, United States; Department of Emergency Medicine, Yale University School of Medicine, New Haven, CT, 06519, United States; Computational Biology and Biomedical Informatics, Yale Graduate School of Arts and Sciences, New Haven, CT, 06510, United States; Biomedical Informatics and Data Science, Yale University School of Medicine, New Haven, CT, 06510, United States; Department of Biostatistics, Yale School of Public Health, New Haven, CT, 06510, United States

**Keywords:** electronic health record, machine learning, physician productivity

## Abstract

**Objective:**

To explore the relationship between ambulatory physician electronic health record (EHR) use characteristics and proxies for physician efficiency.

**Materials and Methods:**

A longitudinal cohort study was conducted to examine physician-month EHR use metadata in 413 US organizations between May 2019 and April 2022. A multi-model machine learning classifier was developed to predict physician efficiency. The main outcomes of the study were physician *efficiency*, measured as the proportion of same-day chart completion by specialty, and *productivity*, measured as daily patient visit volume, both segmented into quintiles.

**Results:**

The study included 218 610 unique physicians with 5 193 385 physician-month observations from 413 organizations with an average chart completion efficiency of 72.9% and 10.8 visits per scheduled day. The primary ML analysis achieved an accuracy of 0.74 in classifying physician-months with high chart completion efficiency and highlighted associations with key features, such as inbox message turnaround time <1.5 days and after-hours documentation <25 min/scheduled day. A secondary analysis achieved an accuracy of 0.84 in classifying physician-months with high visit volumes, indicating that factors such as EHR time outside scheduled hours <4.1 min/visit and clinical review time <3.2 min/visit were associated with higher visit volumes.

**Discussion and Conclusion:**

Implementing specific EHR use measures with distinct thresholds, such as inbox management and after-hours documentation, could help target interventions to enhance productivity, providing actionable insights to create balanced and efficient work environments that improve patient care and reduce EHR time.

## Introduction

Rapid digitization of US healthcare has resulted in a significant electronic health record (EHR) burden for physicians.^1^ In particular, inbox message volume and uncompensated after-hours documentation contribute to physician professional burnout.^2^^,^^3^ Conversely, team efficiency and support can mitigate burnout and increase physician productivity, respectively.^4–6^ Similarly, emerging generative artificial intelligence (AI) solutions may offer relief in the future. For example, AI inbox message management and ambient documentation have great potential to increase physician productivity while also decreasing EHR workload.^7^ However, early results have shown that AI alone is unlikely to resolve EHR burden.^8^^,^^9^ Nevertheless, much work remains to identify specific physician EHR use factors that minimize documentation burden and increase efficiency, facilitating more precise AI integration or other interventions that aim to optimize productivity and mitigate burnout.

Originally developed to meet HIPAA requirements, EHR audit logs capture the duration and details of user interactions, providing valuable insights into healthcare operations.^10^ Recent audit log analyses have begun to identify contributors to physician efficiency and productivity, including note composition patterns and strategies, team-based support, and physician workload differences.^5^^,^^6^^,^^11^^,^^12^ However, the relationships between these factors and chart completion efficiency and visit volume are still unknown. Further, most studies are challenging to implement in practice as they lack clear “targets.” For example, while less after-hours documentation time may indicate greater efficiency and productivity, health system leaders need clear, actionable thresholds to guide operational decisions on deploying resources such as training, staff support, or AI technology to the physicians who need them most.

To address this issue, ML offers a robust approach for identifying features, relationships, and thresholds associated with proxies for ambulatory physician efficiency and productivity, namely chart completion efficiency and visit volume. ML algorithms excel at managing large, complex datasets, identifying non-linear relationships, prioritizing prediction accuracy, and offering scalable models. ML applications to EHR audit logs have demonstrated success in classifying clinical tasks and settings and in exploring correlations between physician interactions and operational outcomes.^13–15^ For example, Lopez et al. utilized an XGBoost algorithm to elucidate the association between EHR use and physician attrition in a longitudinal cohort of physicians, demonstrating that specific EHR use patterns could predict physician departure risk with notable accuracy (receiver operating characteristic area under the curve, ROC AUC) of 0.80.

Therefore, to identify EHR use metrics associated with chart completion efficiency and visit volume in a national data set, we analyzed a 3-year national longitudinal sample of monthly physician EHR use. We aimed to answer the following research question: After controlling for specialty, what EHR use factors are most associated with physician efficiency and productivity, respectively, as defined by: (1) the proportion of charts completed on the same day as the clinic visit and (2) daily patient visit volume? Although this study leveraged a large-scale dataset to characterize physician EHR use and efficiency, it remains constrained by the inherent limitations of structured EHR data and should be interpreted within that context. The measures examined in this study quantify time spent on documentation but do not reflect the quality of or clinical appropriateness of notes, nor the quality of care delivered. In addition, the absence of available Clinical Documentation Improvement (CDI) query data may lead to an underestimation of total documentation workload, particularly for clinicians engaged in extensive CDI-related revisions. We applied an explainable ML method to elucidate interpretable trends and establish meaningful thresholds that can inform interventions aimed at improving physician well-being and productivity interventions for operational leaders.

## Methods

### Study design, setting, participants, data sources

In this longitudinal study, monthly aggregate ambulatory physician EHR use data across all United States-based organizations using the Epic EHR (Epic Systems, Verona, WI) were collected from the national Signal EHR Metadata platform for May 2019 to April 2022. Physician-months with a monthly visit count <1 and specialties with an average weekly visit count <10 (infectious disease only) were excluded, as were surgical specialties due to differences in practice style. Finally, any physician-months missing the outcome variable were excluded. This study was determined to be non-human subjects research due to the de-identified and aggregate nature of the data by the University of California, San Francisco Institutional Review Board.

### Variables and measurements

To ensure that our findings reflect broader trends in EHR use rather than unmeasured variations within specialties, we have controlled for specialty-specific practice patterns by stratifying outcomes by specialty. We selected proxies for ambulatory physician operational efficiency and productivity from available measures within Epic’s Signal national dataset. The *primary outcome of efficiency* was the physician-month’s percentile rank in proportion (percentage) of charts completed on the same day as the corresponding clinical encounter within each specialty, henceforth referred to as chart completion efficiency.^16^^,^^17^ Although chart completion efficiency does not directly assess burnout, prior work linking time to chart closure with clinician burnout^16^ supports chart efficiency as a potential proxy for workflow-related well-being, without implying causality. We define chart completion as signing the encounter note and marking the visit as complete, officially closing the visit while still allowing for later additions, such as lab results. Percentile ranks of each specialty were divided into quintiles based on value thresholds to balance the highly skewed outcome distribution and ensure even distribution across each quintile from the individual level (Figure S1 and Table S3). To identify factors associated with top performance, we stratified analyses by quintile, as this approach best segmented variation in performance (Table S1). This motivated our decision to report results based on the 20th and 80th percentiles. The top 20% (1st quintile, with the highest proportion of same-day chart completion) represented high chart completion efficiency, while the bottom 20% (5th quintile, with the lowest proportion of same-day chart completion) represented low chart completion efficiency, rather than using quintiles.

Visit volume by specialty (defined as average daily visits per scheduled day, ie, days with patient visits) was selected as a *secondary outcome,* as visit volume can help benchmark physician productivity by reducing patient bottlenecks and wait times.^18^^,^^19^ Similar to the primary outcome, the secondary outcome was divided into quintiles based on the percentile rank thresholds of the specialties.

Additional variables were grouped into 4 categories: medical specialty, organization characteristics (geographic region, state, self-reported organization type), panel characteristics (monthly average patient age, patient complexity defined by Healthcare Common Procedure Coding System code across all patients in each physician’s panel),^20^ and number of existing and new patients), and EHR use measurements (including all *68 variables in the primary model and 81 variables in the secondary model* variables available in Epic Signal nationally, such as metrics of active time spent on specific EHR activities like documentation, orders, inbox, and chart review, number of orders, number of characters in notes, and active EHR days during the reporting period, full list in Table S2).

### Data analysis

Chart completion efficiency, visit volume, and EHR use metrics were reported using descriptive statistics and stratified by the top and bottom quintiles of chart completion efficiency. The Kruskal-Wallis test was used to evaluate differences between groups, with statistical significance set at *P* < .05. All variables were examined to determine the need for imputation and normalization. All variables were converted to binary metrics based on missing and non-missing values, and a chi-square test was used to assess whether missingness in the primary and secondary outcome variables occurred at random. We determined a priori that imputation would only be necessary if more than half of the variables were missing at random with *P* > .05. For consistency, visit and note variables were normalized per scheduled day or note.

Separate XGBoost multi-class (softmax) classifier models were built for the primary outcome (chart completion efficiency), and for the secondary outcome (visit volume), using independent preprocessing and feature selection pipelines.^21^ XGBoost was chosen for its robustness to multicollinearity, ability to handle missing data, and strong predictive performance.^22^ Hyperparameter tuning using Optuna (a tree-structured Parzen estimator that adaptively selects promising configurations based on prior evaluations) was performed to maximize the weighted f1 score with 5-fold cross-validation, defined as the average of f1 for each class.^23^^,^^24^ The data were split into training and test sets with an 80%-20% split, stratified by physician to ensure no physician appeared in both the training and test datasets. The performance of XGBoost classifiers was evaluated using confusion matrices and standard ML performance metrics. SHapley Additive exPlanations (SHAP) values were plotted for the top features of each model to identify each model’s key variables most strongly associated with the outcomes, as well as their importance and directionality. All predictions reflect associations identified by the model and should not be interpreted as evidence of causality. SHAP feature dependence was plotted with zero horizontal lines for the top 4 features in each model to visualize how each feature influenced outcome predictions. The zero horizontal lines were used to identify the threshold values for each feature, representing the point where the SHAP values transitioned from positive to negative or vice versa.

## Results

### Sample characteristics and descriptive statistics

**Figure 1.**
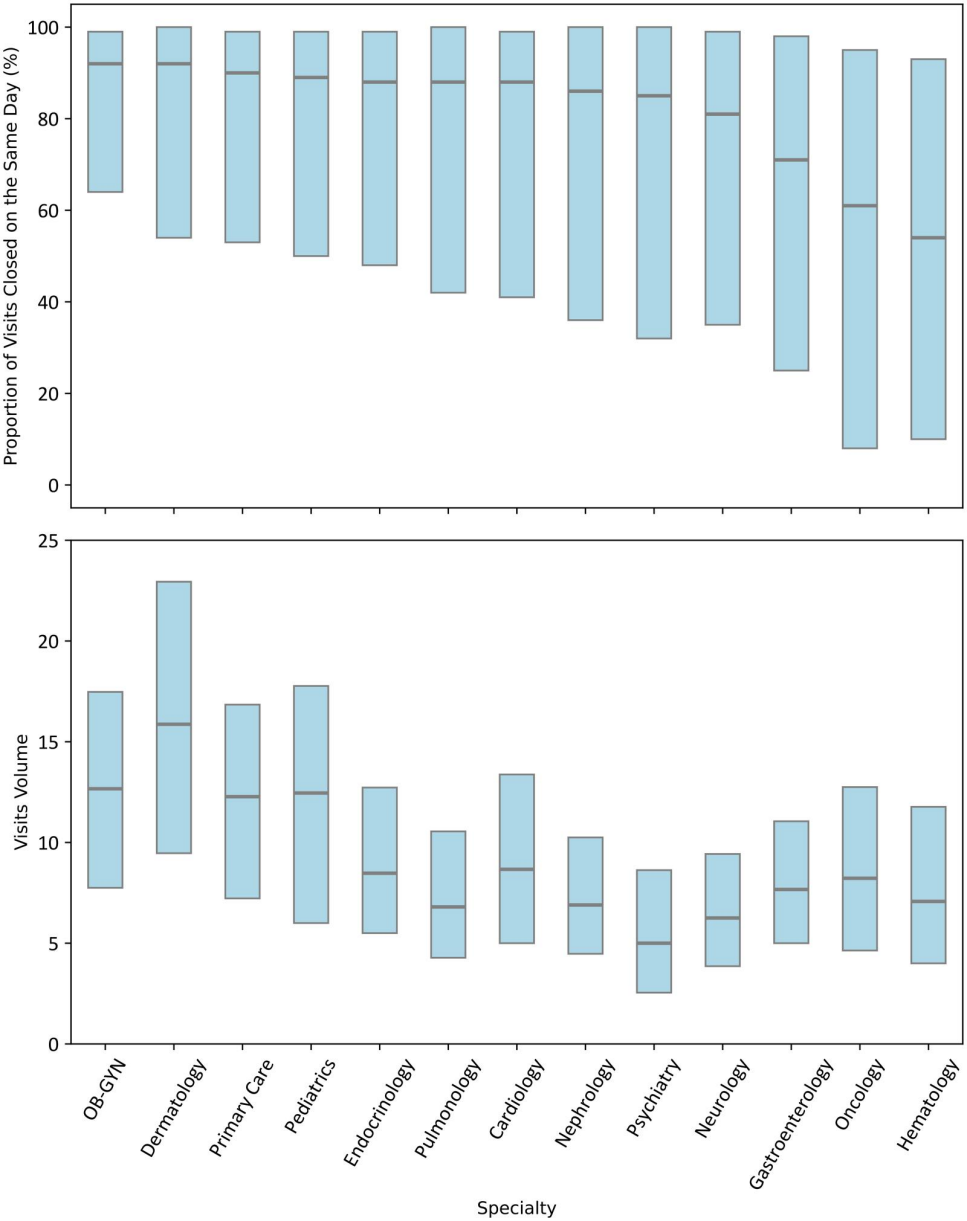
Box plot (median and 20th and 80th percentiles) of the proportion of visits closed on the same day (top panel) and visits per scheduled day (bottom panel) by specialty.

**Figure 2.**
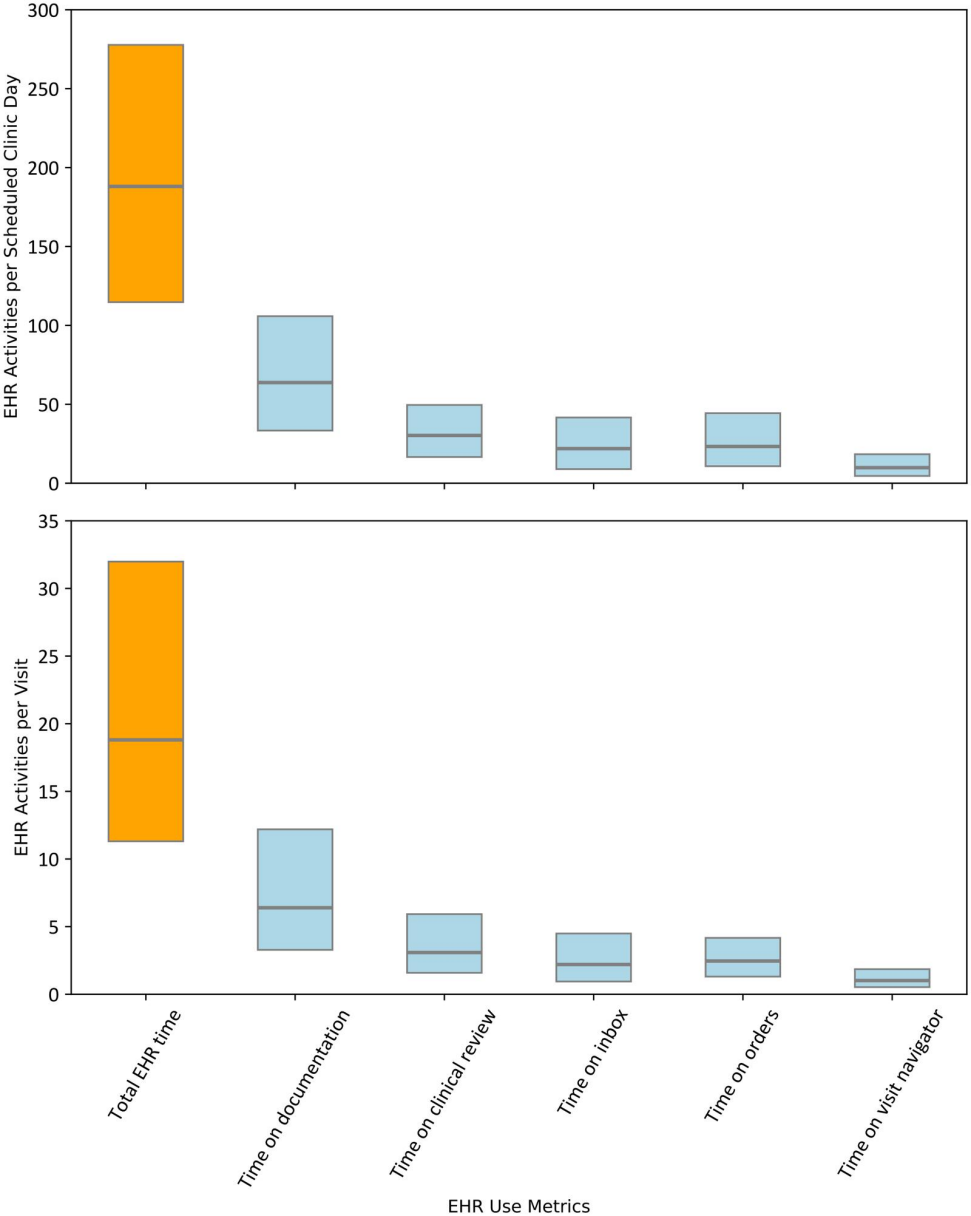
Box plot (median and 20th-80th percentile) displaying time on EHR activities per scheduled clinic day (top panel) and per visit (bottom) panel.

Our final analytic sample included 218 610 unique physicians with 5 193 385 physician-month observations from 413 organizations. Median chart completion efficiency was 88.0% (20th-80th percentile, 44.0-99.0). Physicians had a median of 10.08 (20th-80th percentile 5.41-15.63) visits per scheduled day. There was wide variation within and across specialties (Figure 1): OB-GYN had the highest chart completion efficiency (median 92.0%, 20th-80th percentile 64.0-99.0), and hematology had the lowest (median 54.0%, 20th-80th percentile 10-93). For visit volume, dermatology had the highest median (15.0 visits, 20th-80th percentile 9-22) and psychiatry the lowest (5 visits, 20th-80th percentile 2-8). All specialties except hematology achieved a 100% chart closure rate at the 0.90 threshold and maintained high closure rates at 0.75 (Table S3). Within specialty physician-stratification by high and low chart completion efficiency, the greatest variation was observed in oncology with a 97.5% absolute difference (Table S4, 99% difference, *P* < .001). Physicians spent 199.2 minutes/scheduled day (20th-80th percentile 111.5-276.5, Figure 2) and 24.51 minutes/visit on the EHR (20th-80th percentile 11.1-31.87). Within specialty physician stratification by high and low visit volume, dermatology demonstrated the greatest variation, with an average of 22 additional visits per day (77.6% difference, *P* < .001). Physicians with high chart completion efficiency spent less time per clinic day and per visit on all EHR use metrics measured (*P* < .001 for all), with the largest differences per day in time on documentation (4.9 minutes, 7% less) and the largest difference per visit on orders (0.4 minutes, 79.8% less). More than half of the variables were not missing at random (63% of variables, *P* < .05, Table S2). The average percentage of missing data (mean with 95% CI) was 11.99% (6.75%-17.23%) across 68 variables in the primary model and 12.39% (7.86%-16.91%) across 81 variables in the secondary model.

### Machine learning models

The primary XGBoost multi-class classifier model predicted the proportion of charts completed on the same day with an accuracy of 0.74, precision of 0.49, recall of 0.68, and ROC of 0.81 for high performers. For predicting the bottom quintile of chart completion, the model achieved an accuracy of 0.83, precision of 0.56, recall of 0.64, and ROC of 0.87 (Figures S2 and S3, Table S5).

**Figure 3.**
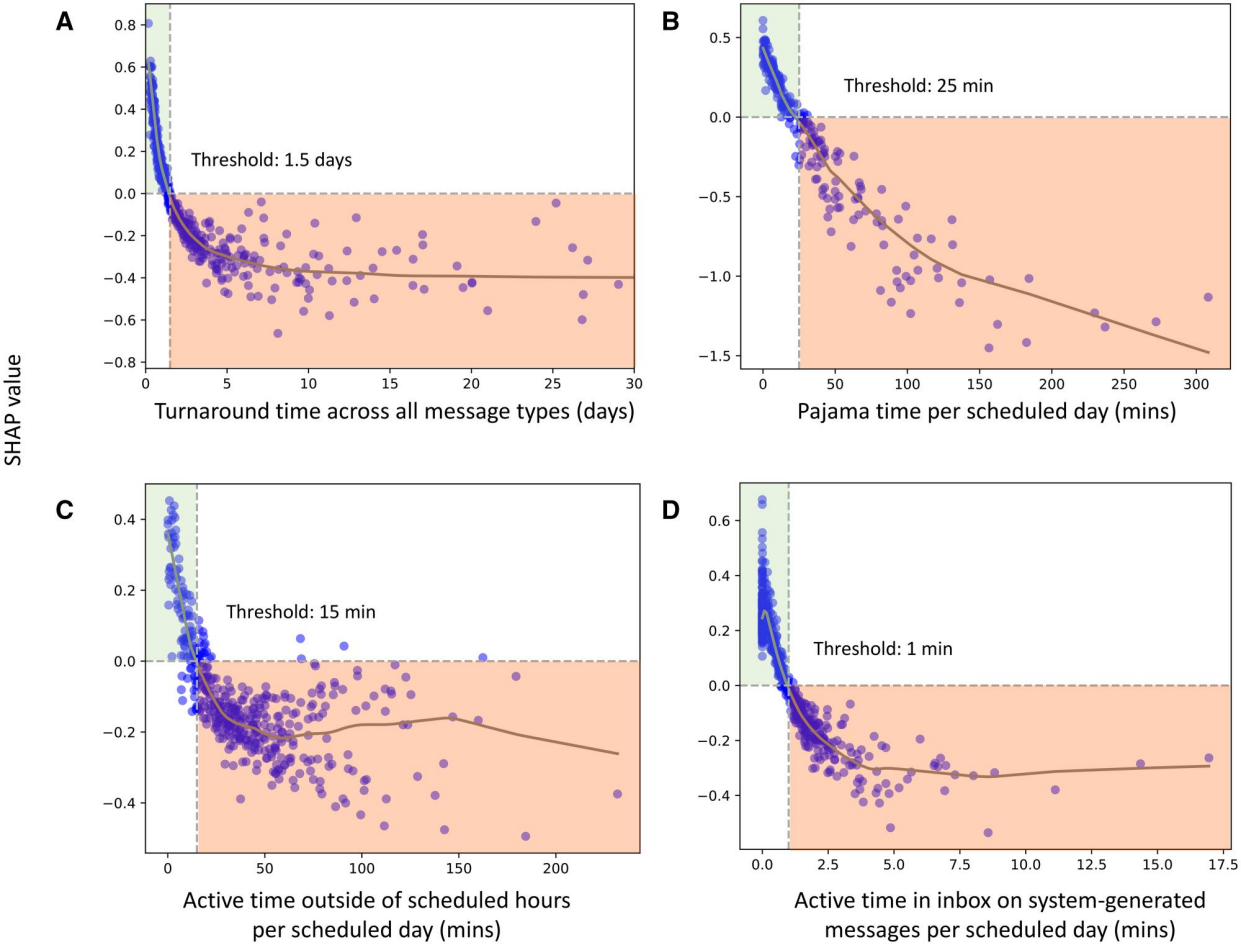
SHAP value estimates for **high chart completion efficiency** for the top four features. Points denote the estimated SHAP value for each physician-month observation. The gray line represents a LOESS estimator of the SHAP value across changes in feature value. The feature value at which the SHAP value crosses 0 is indicated by a vertical dashed line. Positive SHAP values indicate a higher likelihood of high chart completion efficiency, whereas negative SHAP values indicate a lower likelihood.

SHAP values identified these top 10 features for predicting high chart completion efficiency (from most to least important; per scheduled day, unless otherwise indicated): turnaround time across all message types, pajama time (defined as active documentation time outside of 7:00 am-5:20 pm on both weekdays and weekends), active time outside of scheduled hours, inbox time on system-generated messages, number of system-generated messages received, turnaround time for patient medical advice request messages, number of inbox messages marked as “done,” active time in EHR, patient average age, and medication orders pended by others and signed (Figure S4). SHAP feature dependence plots for the top 4 features in predicting high chart completion efficiency illustrate the association between these features and their SHAP values (Figure 3), with all features exhibiting negative associations. Lower turnaround time across all message types, pajama time, EHR time outside of scheduled hours, and inbox time on system-generated messages were all associated with higher chart completion efficiency. Thresholds for prediction in the high chart completion efficiency quintile were turnaround time across all message types <1.5 days, pajama time per scheduled day <25 minutes, active time outside scheduled hours <15 minutes, and time on system-generated messages <1 minute. A plateau effect was observed beyond the following thresholds: turnaround time across all messages (>10 days), time outside of scheduled hours per scheduled day (>80 minutes), and time on system-generated messages per scheduled day (>5 minutes), with association being less pronounced for the latter feature.

The secondary XGBoost regression model predicted visit volume by specialty with an accuracy of 0.84, precision of 0.59, recall of 0.70, and ROC of 0.90 for high performers. For predicting the bottom quintile of chart completion, the model achieved an accuracy of 0.89, precision of 0.72, recall of 0.75, and ROC of 0.94 (Figure S5 and S6, Table S6).

**Figure 4.**
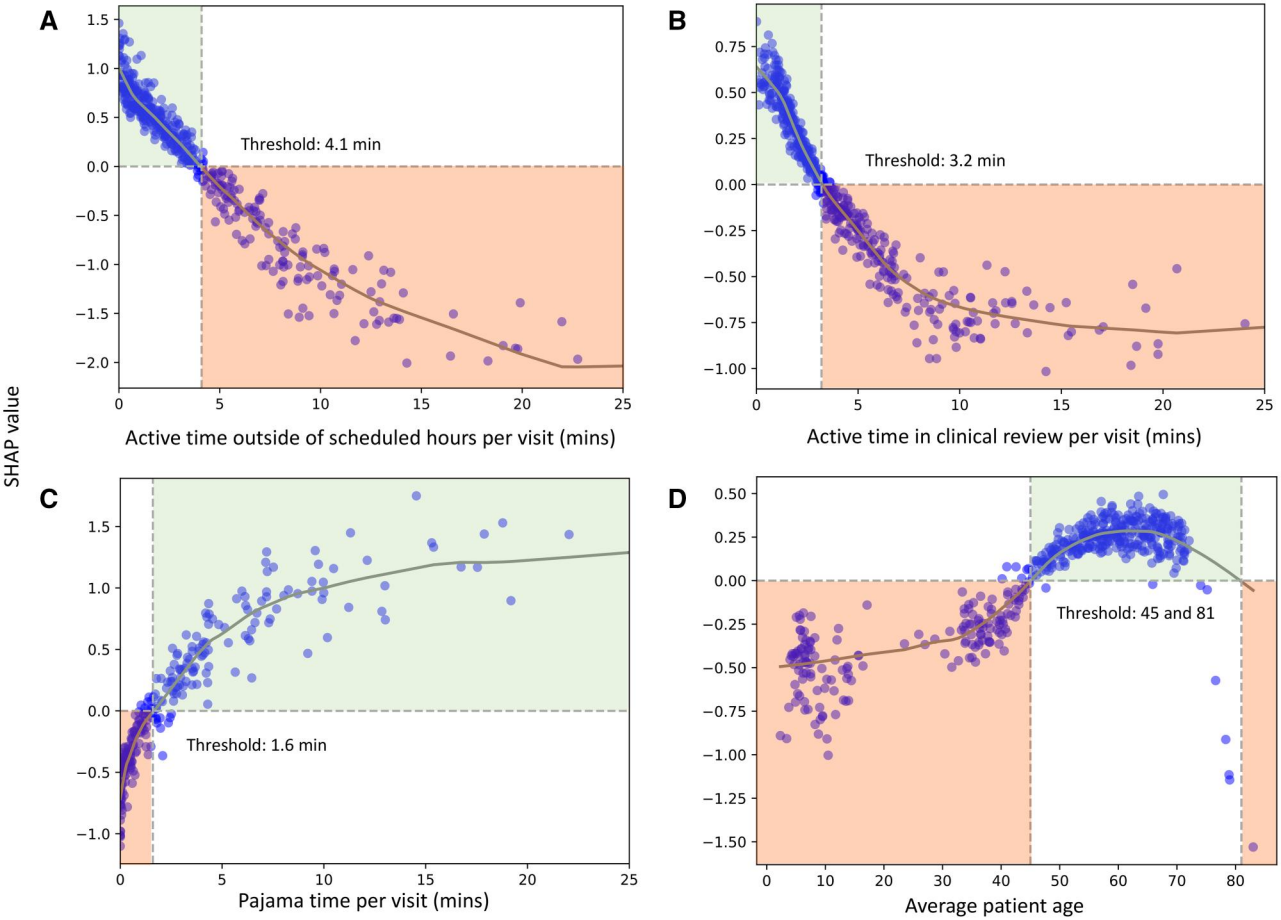
SHAP value estimates for **visit volume per scheduled day** for the top four features. Points denote the estimated SHAP value for each physician-month observation. The gray line represents a LOESS estimator of the SHAP value across changes in feature value. The feature value at which the SHAP value crosses 0 is indicated by a vertical dashed line. Positive SHAP values indicate a higher predicted visit volume per scheduled day, whereas negative SHAP values indicate a lower predicted visit volume per scheduled day.

SHAP values identified these top 10 features for predicting visit volume (from most to least important; per visit, unless otherwise indicated): EHR time outside of scheduled hours, clinical review time (ie, time spent reviewing a patient’s records including notes and results), pajama time, average patient age, time in notes, time outside of scheduled days, inbox time, order time, time on system-generated inbox messages, and visit navigator time (Figure S7). SHAP feature dependence plots for the top 4 features in predicting visit volume illustrate the relationship between each feature and its SHAP values (Figure 4). EHR time outside scheduled hours and clinical review time had negative associations with visit volume, with higher values associated with lower visit volume. Whereas, pajama time and average patient age had positive associations with visit volume, with high values associated with higher visit volume. Threshold values for prediction of high visit volume included (per visit): EHR time outside scheduled hours <4.1 minutes, clinical review time <3.2 minutes, pajama time >1.6 minutes, and average patient age 45-81 years old. A plateau effect was observed beyond the following thresholds (per visit): clinical review time (>8.0 minutes) and pajama time (7.0 minutes).

## Discussion

In this national longitudinal cohort study, explainable ML models accurately identified key aspects of EHR use and discovered actionable thresholds to inform targets for optimizing physician efficiency and productivity. Metrics of inbox time and volume, EHR use outside of scheduled hours, and patient age were identified as having the strongest associations with physician efficiency and productivity. The primary analysis showed that faster inbox message turnaround times (<1.5 days), minimizing after-hours EHR use (<25 min/day), reducing EHR activity outside scheduled hours (<15 min/day), and limiting time spent on system-generated inbox messages (<1 min/day) were associated with higher chart completion efficiency. The secondary analysis revealed that keeping EHR time outside scheduled hours under 4.1 min/visit and clinical review time under 3.2 min/visit was linked to higher visit volumes, while moderate after-hours documentation (>1.6 min/visit) and an older patient demographic were associated with increased productivity. Both analyses provided insights that comprehensive solutions, rather than targeting a single domain, are important.

This study’s strengths include a large sample size, which enhances the power of its results, comprehensive national data, a longitudinal design to account for trends, and consistency with previous literature in visit and EHR use metric values.^25–28^ Furthermore, use of ML methods enables the identification of non-linear relationships while prioritizing prediction accuracy and model scalability, given their capability to handle large and complex datasets more effectively than traditional statistical methods.^29^ Machine learning has previously been successfully applied to EHR audit logs to classify clinical tasks and settings and, more recently, to examine correlations between physician EHR use and operational outcomes.^13–15^^,^^30^ One of the key strengths of explainable ML lies in its ability to identify specific thresholds that can guide operational leaders in pinpointing physicians who may struggle with EHR use, which is associated with efficiency and productivity.^31^ These thresholds, derived from EHR use data, can inform targeted interventions aimed at improving physician workflows. However, these thresholds reflect statistical patterns and should not be interpreted as direct intervention targets without further validation in real-world clinical workflows. This targeted approach contrasts with previous studies that focus on average effects, making it difficult to identify individual physicians or translate evidence into practice. By providing concrete, actionable insights, explainable ML could enable tailored resource deployment, optimizing support for those most in need, and enhancing overall operational performance.

One important methodological consideration in this study is the recognition that efficiency and workload metrics differ substantially by specialty. This known variability motivated our stratification of both primary (chart completion efficiency) and secondary (visit volume) outcomes into specialty-specific quantiles to standardize comparisons across clinical contexts and workflows. The threshold test results (Tables S3 and S4) further supported this approach, showing that while overall chart completion efficiency was high across specialties, there remained meaningful variation likely reflecting differences in documentation needs and workflow structure. Similarly, visit volume patterns varied by specialty, underscoring that efficiency and productivity must be interpreted within their specialty context.

Our findings build on recent audit log analyses that have identified contributors to physician efficiency by providing new insights, including precise thresholds for productivity. A recent local analysis of general internal medicine ambulatory physicians,^5^ along with a national longitudinal ambulatory physician cohort study,^6^ demonstrated that increased team support and the adoption of team-based documentation increased productivity and visit volumes while reducing time spent on documentation and EHR-related tasks. While our top features did not directly reflect team support, they highlight opportunities for documentation and inbox support to sustain high productivity. A recent national study found an association between higher after-hours documentation and burnout, emphasizing the potential of EHR-based smart tools to reduce this burden.^11^ Additionally, a recent national study emphasized the value of transparent, data-driven discussions between health system leaders and physicians regarding work-hour expectations.^12^ Our findings align with this, indicating that uncompensated after-hours documentation is not associated with higher productivity and may be associated with lower chart completion efficiency.

Our findings underscore the importance of streamlining clinical and EHR workflows to optimize both efficiency and clinician well-being, as prolonged EHR work outside scheduled hours is associated with reduced efficiency and may contribute to increased strain. Our study reveals that EHR use patterns linked to top performance include optimized inbox management and after-hours documentation to enhance productivity and potentially mitigate subsequent burnout. Shorter message turnaround times for and lower pajama time were associated with higher same-day chart completion, suggesting that after-hours work patterns may reflect underlying workflow strain during scheduled hours rather than inefficiency per se. Moreover, efficient management of older patient visits was associated with higher visit volumes, suggesting that strategies for efficiently managing complex cases could improve productivity. These findings underscore the need for targeted interventions to optimize team support and streamline physician workflows, ultimately alleviating burnout and improving patient outcomes. Additionally, these findings may reflect multiple, non-mutually exclusive pathways. One potential explanation is that completing EHR documentation during the patient visit may be more efficient than deferring this work to later in the day or after hours. Alternatively, clinicians who reduce time spent on other EHR tasks, such as managing inbox messages, may have more available time to complete documentation efficiently. Another possibility is that some individuals possess generalizable efficiency workflows or skills that extend across various EHR tasks.

Our findings align with known healthcare operational challenges and offer actionable insights for intervention.^32^ Chart completion efficiency was associated with efficient inbox responses and minimal after-hours work, while higher on-site EHR use and lower pajama time per visit may indicate more balanced documentation workflows. Older patient age may also affect productivity due to increased chart review time. For clinicians, the implications are clear: reducing after-hours EHR burden and optimizing in-clinic EHR workflows may improve operational efficiency and, by mitigating EHR burden, support clinician well-being.^33^ Operational leaders could use these thresholds to guide interventions, such as team-based documentation and scribe programs, which could reduce charting time and improve inbox management. For instance, inbox pools or automated responses may help manage message volume and potentially free up time for other clinical tasks, such as chart completion. Meanwhile, strategic scheduling and ambient listening technology may help balance workload and minimize after-hours work. Identifying physicians who struggle with these metrics could enable tailored support, ultimately optimizing workload and resource allocation. Predictions based on these models could help adjust appointment length, frequency, and structure to further balance patient complexity and productivity in the future.

Future work could apply similar analyses in other settings, such as inpatient care and emergency departments, and explore variability by practice type or region to determine if the identified thresholds are consistent with other productivity metrics, like RVUs. Furthermore, given the complexity and difficulty of changing EHR systems, it would be beneficial to investigate the chart completion efficiency and visit volume with different EHR vendor products. Longitudinal studies could examine the long-term effects of optimized EHR use patterns on physician burnout and patient outcomes. Focused analyses on the identified thresholds are essential to evaluate how interventions, such as scribes, virtual scribes, and ambient listening technology, impact efficiency, productivity, and physician well-being. This evaluation could inform implementation strategies, cost-effectiveness analyses, and facilitate the widespread adoption of optimized interventions. In practice, while both chart closure efficiency and visit volume productivity are desirable operational goals, they may occasionally conflict. Leaders must weigh trade-offs and select strategies and interventions that best align with their health system’s management style and overarching goals. Future work should incorporate documentation quality metrics or CDI query data to provide a more comprehensive view of efficiency that captures both the speed and quality of EHR documentation.

### Limitations

While this study leveraged a large, national dataset to characterize physician EHR use and efficiency, several important limitations should be recognized when interpreting the results and key findings. First, audit logs do not capture all aspects of physician work, potentially missing other contributors to efficiency and productivity that take place outside of the EHR. Second, although the data are longitudinal, the observational design limits our ability to establish causality between EHR use patterns and physician efficiency outcomes. Third, our outcomes do not capture the quality of care or clinical effectiveness, and should not be interpreted as proxies for patient-centered outcomes. Fourth, using data from Epic alone may limit the generalizability of our results, as they may not reflect all practice environments. However, Epic is the largest EHR vendor in the United States,^34^ and is used by a broad range of health systems, including academic, safety-net, and community organizations. Fifth, because our dataset did not include note content or CDI query rates, we were unable to assess documentation quality directly. Consequently, our analysis focuses on chart completion time as a measure of documentation quantity rather than quality. The absence of CDI data may lead to underestimation of total documentation workload, particularly for clinicians engaged in extensive CDI-related revisions. Sixth, quintile-based segmentation may not be the optimal approach for all scenarios. Seventh, although we identified key efficiency thresholds, the study did not investigate the underlying reasons for variability in EHR use across different specialties or individual physicians. Finally, correlated features in the model may still complicate the interpretation of individual variable contributions, which can distribute importance across related variables.

## Conclusions

Our study illuminates new details on the intricate relationship between EHR use patterns and physician efficiency, highlighting critical thresholds that could inform targeted interventions.

Streamlining EHR processes and team-based support, as well as adopting supportive technologies to address inbox volume, after-hours documentation, and complex case management, could enhance physician productivity and efficiency. These findings offer policymakers and administrators actionable insights to optimize clinical operations and foster a more balanced, efficient work environment that enhances patient care.

## Supplementary Material

ooag018_Supplementary_Data

## Data Availability

The data underlying this article cannot be shared publicly due to privacy and licensing restrictions. All data used in the study are available upon reasonable request from the data provider, Epic Systems.
