# Supplementary material for: Electronic health record use factors linked to efficiency and productivity: an explainable machine learning analysis
Source: JAMIA Open. 2026 Feb 25;9(1):ooag018. doi: 10.1093/jamiaopen/ooag018 (PMC12936052; doi:10.1093/jamiaopen/ooag018)
Supplement: ooag018_Supplementary_Data [file ooag018_supplementary_data.docx]

**Supplementary Materials**

1. **Supplemental Figure 1.** Data distribution of the representative specialty for the primary outcome with threshold ranges by quintile indicated using dashed lines
2. **Supplemental Table 1.** Threshold values for each specialty in each quintile
3. **Supplemental Table 2.**
   1. chi-square test result for chart completion efficiency (top and bottom quantile)
   2. chi-square test result for visit volume (top and bottom quantile)
4. **Supplemental Table 3.** Threshold test values for each specialty for primary outcome
5. **Supplemental Table 4.** Descriptive statistics for chart completion efficiency, visit volume, and EHR use metrics with stratification by specialty as well as high and low chart completion efficiency and visit volume.
6. **Supplemental Figure 2.** Confusion matrix for XGBoost multi-class classifier model predicting the proportion of charts completed.
7. **Supplemental Figure 3.** ROC Curves for each class for XGBoost multi-class classifier model predicting the proportion of charts completed.
8. **Supplemental Table 5.** Evaluation metrics of XGBoost multi-class classifier model performance in predicting proportion of charts completed.
9. **Supplemental Figure 4.** SHAP summary plot for top 10 features in predicting high same day chart completion.
10. **Supplemental Figure 5.** **.** Confusion matrix for XGBoost multi-class classifier model predicting the value visit volume per scheduled day.
11. **Supplemental Figure 6.** ROC Curves for each class for XGBoost multi-class classifier model predicting visit volume per scheduled day.
12. **Supplemental Table 6.**  Evaluation metrics of XGBoost multi-class classifier model performance in predicting visit volume per scheduled day
13. **Supplemental Figure 7.** SHAP summary plot for top 10 features in predicting visit volume per scheduled day

**Supplemental Figure 1**: Data distribution of the representative specialty for the primary outcome with threshold ranges by quintile indicated using dashed lines

**Supplemental Table 1:** Threshold values for each specialty in each quintile

|  | Primary outcome threshold | | | | Secondary outcome threshold | | | |
| --- | --- | --- | --- | --- | --- | --- | --- | --- |
| Specialty | 1st Quintile (20%) | 2nd Quintile (40%) | 3rd Quintile (60%) | 4th Quintile (80%) | 1st Quintile (20%) | 2nd Quintile (40%) | 3rd Quintile (60%) | 4th Quintile (80%) |
| Cardiology | 99 | 94 | 78 | 41 | 13.38 | 10.00 | 7.43 | 5.00 |
| Dermatology | 100 | 96 | 85 | 54 | 22.94 | 18.06 | 13.80 | 9.47 |
| Endocrinology | 99 | 94 | 79 | 48 | 12.73 | 9.64 | 7.47 | 5.50 |
| Gastroenterology | 98 | 84 | 57 | 25 | 11.05 | 8.63 | 6.77 | 5.00 |
| Hematology | 93 | 69 | 39 | 10 | 11.77 | 8.27 | 6.00 | 4.00 |
| Nephrology | 100 | 93 | 74 | 36 | 10.25 | 7.78 | 6.00 | 4.47 |
| Neurology | 99 | 90 | 69 | 35 | 9.43 | 7.10 | 5.44 | 3.86 |
| OB-GYN | 99 | 96 | 87 | 64 | 17.47 | 14.09 | 11.21 | 7.75 |
| Oncology | 95 | 75 | 44 | 8 | 12.75 | 9.50 | 7.00 | 4.64 |
| Pediatrics | 99 | 95 | 80 | 50 | 17.77 | 14.11 | 10.67 | 6.00 |
| Primary Care | 99 | 95 | 82 | 53 | 16.84 | 13.67 | 10.80 | 7.22 |
| Psychiatry | 100 | 93 | 71 | 32 | 8.63 | 6.00 | 4.16 | 2.55 |
| Pulmonology | 100 | 94 | 78 | 42 | 10.55 | 7.80 | 6.00 | 4.28 |

**Supplemental Table 2.**

1. chi-square test result for chart completion efficiency (top and bottom quantile)

|  | **Chart completion efficiency** | | | |
| --- | --- | --- | --- | --- |
|  | **Top quintile** | | **Bottom quintile** | |
| **Variables** | **Chi-Square** | **p-value** | **Chi-Square** | **p-value** |
| Region | 18287.91 | 0.00 | 26571.78 | 0.00 |
| Average Patient Age | 73436.78 | 0.00 | 64709.92 | 0.00 |
| Average Number of Problems on Problem List | 48175.23 | 0.00 | 39740.21 | 0.00 |
| Average Number of Characters per Note | 1121951.53 | 0.28 | 1161463.38 | 0.00 |
| Average Number of Characters per Progress Note | 1244257.10 | 0.01 | 1281503.25 | 0.00 |
| Number of SmartPhrases Created | 24203.27 | 0.00 | 34038.47 | 0.00 |
| Number of SmartPhrases Shared | 25694.45 | 0.00 | 31117.37 | 0.00 |
| Average Turnaround Time Across All Message Types per Day | 276794.00 | 0.00 | 227833.39 | 0.00 |
| Average Turnaround Time for Patient Medical Advice Request Messages per Day | 212429.69 | 0.00 | 161562.51 | 0.00 |
| State | 23075.12 | 0.00 | 28398.61 | 0.00 |
| Academic Organization (Yes/No) | 21471.72 | 0.00 | 18629.94 | 0.00 |
| Catholic Organization (Yes/No) | 2588.67 | 0.00 | 618.70 | 0.00 |
| Community Hospital (Yes/No) | 232.05 | 0.00 | 514.82 | 0.00 |
| Pediatric Only Organization (Yes/No) | 4797.54 | 0.00 | 15393.69 | 0.00 |
| Research Organization (Yes/No) | 5390.76 | 0.00 | 7981.08 | 0.00 |
| Safety Net Organization (Yes/No) | 3475.84 | 0.00 | 5517.73 | 0.00 |
| Teaching Organization (Yes/No) | 8436.03 | 0.00 | 10886.14 | 0.00 |
| Visit per Scheduled Day | 140059.92 | 0.00 | 127598.84 | 0.00 |
| Organization Size (Number of Physician-Month) | 194355.96 | 0.00 | 244439.13 | 0.00 |
| Number of Characters using SmartBlock from a Macro per Note | 1257921.75 | 0.00 | 1211246.34 | 1.00 |
| Number of Characters using Voice Recognition per Note | 1652603.93 | 0.00 | 1583537.68 | 1.00 |
| Number of Characters using Copy/Paste per Note | 1762349.79 | 1.00 | 1725934.16 | 1.00 |
| Number of Characters using SmartBlock from a Template per Note | 1983295.14 | 0.00 | 1865693.56 | 1.00 |
| Number of Characters using SmartBlock per Note | 1885276.90 | 0.00 | 1715289.05 | 1.00 |
| Number of Characters using SmartTest per Note | 2099167.07 | 0.00 | 1974806.15 | 1.00 |
| Number of Characters using SmartList per Note | 2150608.90 | 0.00 | 1896928.13 | 1.00 |
| Number of Characters using Copy per Note | 2780763.20 | 1.00 | 2648826.83 | 1.00 |
| Number of Characters using SmartPhrase per Note | 3583322.43 | 1.00 | 3396283.66 | 1.00 |
| Number of Characters using SmartLink per Note | 3984121.79 | 1.00 | 3846817.19 | 1.00 |
| Number of Manual Characters per Note | 3522516.07 | 1.00 | 3359197.65 | 1.00 |
| Time in Inox on the "Send Patient Message" Page per Scheduled Day | 269378.13 | 0.00 | 251951.80 | 1.00 |
| Time in Inbox on the Alert Bar per Scheduled Day | 34469.06 | 0.00 | 36397.95 | 0.00 |
| Time in Inbox on the Home Page per Scheduled Day | 186379.08 | 0.00 | 145234.44 | 1.00 |
| Pajama Time per Scheduled Day | 791883.03 | 1.00 | 1121466.73 | 0.00 |
| Time in Inbox on Patient Medical Advice Request Messages (PMAR) per Scheduled Day | 200702.64 | 0.00 | 189321.53 | 0.00 |
| Number of Patient Medical Advice Request Messages per Scheduled Day | 31179.37 | 0.00 | 37287.87 | 0.00 |
| Time in Inbox on Messages with No Specific Subtype per Scheduled Day | 489087.16 | 0.00 | 472231.13 | 0.00 |
| Number of Pool Messages the Provider Completed per Scheduled Day | 34622.24 | 0.00 | 28078.64 | 0.00 |
| Number of Inbox Messages Marked as "Done" per Scheduled Day | 133757.35 | 0.00 | 93645.64 | 0.00 |
| Time in Visit Navigator per Scheduled Day | 627279.27 | 0.00 | 686440.04 | 0.00 |
| Medication Orders Pended by Others and Signed per Scheduled Day | 51907.56 | 0.00 | 37589.31 | 0.00 |
| Medication Orders Started and Signed per Scheduled Day | 72589.17 | 0.00 | 154985.89 | 0.00 |
| Non-medication Orders Started and Signed per Scheduled Day | 93557.27 | 0.00 | 145596.91 | 0.00 |
| Non-medication Orders Pended by Others and Signed per Scheduled Day | 61696.29 | 0.00 | 53789.25 | 0.00 |
| Time in Inbox on Patient Messages per Scheduled Day | 234482.57 | 0.00 | 219221.98 | 0.34 |
| Time in Inbox on Non-PMAR Patient Messages per Scheduled Day | 37414.17 | 0.00 | 33094.94 | 0.00 |
| Time in Inbox on Team Members Messages per Scheduled Day | 409960.83 | 0.00 | 430338.47 | 0.00 |
| Time in Inbox on CareEverywhere Messages per Scheduled Day | 66163.46 | 0.00 | 62865.51 | 0.00 |
| Time in Inbox on Results per Scheduled Day | 396242.01 | 0.00 | 360622.22 | 0.00 |
| Time in Inbox on Precription and Medication-related Messages per Scheduled Day | 160622.34 | 0.00 | 170596.78 | 0.00 |
| Time in Inbox on System-generated Messages per Scheduled Day | 189374.96 | 0.00 | 262598.49 | 0.00 |
| Time in Inbox on Custom Messages Types per Scheduled Day | 99866.45 | 0.00 | 87520.83 | 0.00 |
| Number of Inbox Messages Received per Scheduled Day | 162284.66 | 0.00 | 107899.53 | 0.00 |
| Number of Patient Messages Received per Scheduled Day | 54457.04 | 0.00 | 38728.25 | 0.00 |
| Number of Non-PMAR Patient Messages Received per Scheduled Day | 10367.92 | 0.00 | 6492.57 | 0.00 |
| Number of Team Members Messages Received per Scheduled Day | 107416.86 | 0.00 | 75319.62 | 0.00 |
| Number of CareEverywhere Messages Received per Scheduled Day | 25710.65 | 0.00 | 21033.71 | 0.00 |
| Number of Results Messages Received per Scheduled Day | 77083.13 | 0.00 | 51112.50 | 0.00 |
| Number of Prescription and Medication-related Messages Received per Scheduled Day | 48408.40 | 0.00 | 55688.45 | 0.00 |
| Number of System-generated Messages Received per Scheduled Day | 181618.95 | 0.00 | 237218.85 | 0.00 |
| Number of Custom Messages Types Received per Scheduled Day | 31492.40 | 0.00 | 29428.53 | 0.00 |
| Time Outside Scheduled Hours per Scheduled Day | 1148683.37 | 1.00 | 1298831.34 | 0.00 |
| Time in Inbox per Scheduled Day | 982515.51 | 0.00 | 970791.91 | 0.00 |
| Time in Documentation per Scheduled Day | 1563940.01 | 1.00 | 1724110.13 | 0.00 |
| Time in Orders per Scheduled Day | 1017678.72 | 0.00 | 1109228.29 | 0.00 |
| Time in Clinical Review per Scheduled Day | 1014573.68 | 0.00 | 1090349.95 | 0.00 |
| Time in Schedule per Scheduled Day | 591081.29 | 0.00 | 544716.99 | 0.00 |
| Time in EHR per Scheduled Day | 2359415.50 | 1.00 | 2537649.64 | 0.00 |

1. chi-square test result for visit volume (top and bottom quantile)

|  | **Visit volume** | | | |
| --- | --- | --- | --- | --- |
|  | **Top quintile** | | **Bottom quintile** | |
| **Variables** | **Chi-Square** | **p-value** | **Chi-Square** | **p-value** |
| Region | 728.70 | 0.00 | 912.87 | 0.00 |
| Average Patient Age | 9603.45 | 0.00 | 9591.30 | 0.00 |
| Average Number of Problems on Problem List | 5471.77 | 0.06 | 5548.90 | 0.01 |
| Proportion of Visits Closed on the Same Day | 166.35 | 0.00 | 169.59 | 0.00 |
| Proportion of Visits Closed within 1-2 Days | 176.59 | 0.00 | 143.66 | 0.00 |
| Proportion of Visits Closed within 3-4 Days | 120.63 | 0.08 | 113.66 | 0.17 |
| Proportion of Visits Closed within 5-6 Days | 93.66 | 0.66 | 118.28 | 0.10 |
| Proportion of Visits Closed within 7+ Days | 180.77 | 0.00 | 138.03 | 0.01 |
| Average Number of Characters per Note | 1122125.41 | 0.24 | 1124415.37 | 0.01 |
| Average Number of Characters per Progress Note | 1238580.11 | 0.90 | 1243982.61 | 0.02 |
| Number of SmartPhrases Created | 7360.13 | 0.00 | 6973.66 | 0.00 |
| Number of SmartPhrases Shared | 8041.47 | 0.00 | 7573.72 | 0.00 |
| Average Turnaround Time Across All Message Types per Day | 43135.56 | 0.98 | 44530.82 | 0.00 |
| Average Turnaround Time for Patient Medical Advice Request Messages per Day | 23683.01 | 0.13 | 23605.79 | 0.22 |
| Time in Clinical Review per Visit | 3246869.88 | 0.00 | 3228511.33 | 1.00 |
| Time in Documentation per Visit | 3725610.74 | 0.02 | 3708012.20 | 1.00 |
| Time in Inbox per Visit | 3098755.83 | 0.05 | 3084787.45 | 1.00 |
| Time in Orders per Visit | 2956924.32 | 0.00 | 2935815.95 | 1.00 |
| Time Outside Scheduled Hours per Visit | 3311431.36 | 0.01 | 3298888.60 | 0.99 |
| State | 5055.06 | 0.00 | 4785.27 | 0.00 |
| Academic Organization (Yes/No) | 382.49 | 0.00 | 31.64 | 0.00 |
| Catholic Organization (Yes/No) | 70.52 | 0.00 | 372.70 | 0.00 |
| Community Hospital (Yes/No) | 33.14 | 0.00 | 512.34 | 0.00 |
| Pediatric Only Organization (Yes/No) | 85.88 | 0.00 | 411.05 | 0.00 |
| Research Organization (Yes/No) | 141.09 | 0.00 | 143.67 | 0.00 |
| Safety Net Organization (Yes/No) | 490.77 | 0.00 | 95.70 | 0.00 |
| Teaching Organization (Yes/No) | 314.48 | 0.00 | 418.41 | 0.00 |
| Organization Size (Number of Physician-Month) | 26958.41 | 0.00 | 27239.06 | 0.00 |
| Number of Characters using SmartBlock from a Macro per Note | 1254174.93 | 0.10 | 1250425.53 | 0.86 |
| Number of Characters using Voice Recognition per Note | 1641680.49 | 0.45 | 1641264.98 | 0.54 |
| Number of Characters using Copy/Paste per Note | 1772038.93 | 0.15 | 1769523.74 | 0.62 |
| Number of Characters using SmartBlock from a Template per Note | 1969254.35 | 0.06 | 1964609.55 | 0.79 |
| Number of Characters using SmartBlock per Note | 1848901.39 | 0.01 | 1840678.07 | 0.96 |
| Number of Characters using SmartTest per Note | 2070272.08 | 0.17 | 2062256.59 | 1.00 |
| Number of Characters using SmartList per Note | 2095826.05 | 0.00 | 2079768.48 | 1.00 |
| Number of Characters using Copy per Note | 2804137.14 | 0.06 | 2791775.27 | 1.00 |
| Number of Characters using SmartPhrase per Note | 3613059.83 | 0.03 | 3596830.17 | 1.00 |
| Number of Characters using SmartLink per Note | 4039662.88 | 0.13 | 4033189.28 | 0.88 |
| Number of Manual Characters per Note | 3591655.08 | 0.00 | 3573664.70 | 1.00 |
| Time in Inox on the "Send Patient Message" Page per Visit | 799087.20 | 0.38 | 793134.28 | 1.00 |
| Time in Inbox on the Alert Bar per Visit | 186812.67 | 0.32 | 184659.42 | 1.00 |
| Time in Inbox on the Home Page per Visit | 400378.11 | 1.00 | 409173.14 | 0.00 |
| Pajama Time per Visit | 1948981.63 | 0.39 | 1944871.42 | 0.97 |
| Time in Inbox on Patient Medical Advice Request Messages (PMAR) per Visit | 1057318.90 | 0.02 | 1046520.31 | 1.00 |
| Number of Patient Messages Received per Visit | 147676.83 | 0.05 | 143952.66 | 1.00 |
| Time Outside Scheduled Days per Visit | 3047313.28 | 0.04 | 3036746.02 | 0.99 |
| Time in Inbox on Messages with No Specific Subtype per Visit | 1928623.47 | 1.00 | 1949613.60 | 0.00 |
| Number of Pool Messages the Provider Completed per Visit | 107881.84 | 0.00 | 103076.73 | 1.00 |
| Number of Inbox Messages Marked as "Done" per Visit | 568321.04 | 0.00 | 555086.75 | 1.00 |
| Time in Visit Navigator per Visit | 2492601.42 | 0.00 | 2471493.12 | 1.00 |
| Medication Orders Pended by Others and Signed per Visit | 111464.13 | 0.00 | 109901.54 | 0.72 |
| Medication Orders Started and Signed per Visit | 193886.51 | 0.00 | 189516.60 | 1.00 |
| Non-medication Orders Started and Signed per Visit | 346710.42 | 0.00 | 336030.59 | 1.00 |
| Non-medication Orders Pended by Others and Signed per Visit | 214214.68 | 0.00 | 207223.14 | 1.00 |
| Time in Inbox on Patient Messages per Visit | 1081329.31 | 1.00 | 1096870.07 | 0.88 |
| Time in Inbox on Non-PMAR Patient Messages per Visit | 147099.84 | 1.00 | 161565.23 | 0.00 |
| Time in Inbox on Team Members Messages per Visit | 1828169.16 | 1.00 | 1876597.02 | 0.00 |
| Time in Inbox on CareEverywhere Messages per Visit | 324018.14 | 1.00 | 327010.99 | 1.00 |
| Time in Inbox on Results per Visit | 1630354.69 | 1.00 | 1626880.12 | 1.00 |
| Time in Inbox on Prescription and Medication-related Messages per Visit | 857644.57 | 1.00 | 881317.17 | 0.01 |
| Time in Inbox on System-generated Messages per Visit | 1038620.23 | 1.00 | 1058288.68 | 0.01 |
| Time in Inbox on Custom Messages Types per Visit | 445869.24 | 1.00 | 445829.11 | 1.00 |
| Number of Inbox Messages Received per Visit | 572673.14 | 0.00 | 559605.49 | 1.00 |
| Number of Patient Messages Received per Visit | 148497.18 | 0.12 | 144507.84 | 1.00 |
| Number of Non-PMAR Patient Messages Received per Visit | 17515.49 | 1.00 | 19178.69 | 0.00 |
| Number of Team Members Messages Received per Visit | 341416.23 | 0.00 | 331505.25 | 1.00 |
| Number of CareEverywhere Messages Received per Visit | 109442.15 | 0.00 | 105047.85 | 1.00 |
| Number of Results Messages Received per Visit | 201850.24 | 0.00 | 195821.35 | 1.00 |
| Number of Prescription and Medication-related Messages Received per Visit | 176945.38 | 0.28 | 177627.30 | 0.04 |
| Number of System-generated Messages Received per Visit | 267186.49 | 0.01 | 262188.24 | 1.00 |
| Number of Custom Messages Types Received per Visit | 86751.21 | 0.05 | 81490.82 | 1.00 |
| Percent of HCPCS 99201 Visits to Total HCPCS 99201-205 Visits | 3558.98 | 0.15 | 3668.36 | 0.01 |
| Percent of HCPCS 99202 Visits to Total HCPCS 99201-205 Visits | 9337.08 | 0.18 | 9079.35 | 0.84 |
| Percent of HCPCS 99203 Visits to Total HCPCS 99201-205 Visits | 11961.02 | 0.00 | 11218.47 | 0.94 |
| Percent of HCPCS 99204 Visits to Total HCPCS 99201-205 Visits | 10388.86 | 0.00 | 9636.50 | 0.91 |
| Percent of HCPCS 99205 Visits to Total HCPCS 99201-205 Visits | 4531.48 | 0.08 | 4077.73 | 1.00 |
| Percent of HCPCS 99211 Visits to Total HCPCS 99211-215 Visits | 14120.82 | 0.13 | 14270.04 | 0.02 |
| Percent of HCPCS 99212 Visits to Total HCPCS 99211-215 Visits | 28670.66 | 0.00 | 26774.62 | 1.00 |
| Percent of HCPCS 99213 Visits to Total HCPCS 99211-215 Visits | 60036.83 | 0.00 | 57595.79 | 0.99 |
| Percent of HCPCS 99214 Visits to Total HCPCS 99211-215 Visits | 60014.76 | 0.00 | 58674.23 | 0.43 |
| Percent of HCPCS 99215 Visits to Total HCPCS 99211-215 Visits | 28662.52 | 0.05 | 28268.40 | 0.51 |

**Supplemental Table 3.** Threshold test values for each specialty for primary outcome

| Physician-month | |  |  |  |  |  |  |  |  |  |  |  |  |  |
| --- | --- | --- | --- | --- | --- | --- | --- | --- | --- | --- | --- | --- | --- | --- |
|  | Percentile | Primary Care (%) | Pediatrics (%) | OB-GYN (%) | Cardiology (%) | Oncology (%) | Psychiatry (%) | Neurology (%) | Gastroenterology (%) | Pulmonology (%) | Nephrology (%) | Endocrinology (%) | Dermatology (%) | Hematology (%) |
| top 10% | 0.90 | 100 | 100 | 100 | 100 | 99 | 100 | 100 | 100 | 100 | 100 | 100 | 100 | 99 |
| top 25% | 0.75 | 99 | 99 | 99 | 98 | 91 | 99 | 98 | 96 | 99 | 99 | 98 | 99 | 89 |
| top 50% | 0.50 | 90 | 89 | 92 | 88 | 60 | 85 | 80 | 72 | 88 | 86 | 88 | 92 | 54 |
| Low 30% | 0.30 | 70 | 68 | 78 | 63 | 25 | 53 | 53 | 41 | 62 | 57 | 66 | 72 | 24 |
|  | 0.25 | 62 | 60 | 72 | 53 | 16 | 43 | 44 | 33 | 52 | 47 | 58 | 64 | 17 |
|  | 0.15 | 42 | 40 | 55 | 27 | 1 | 19 | 24 | 15 | 30 | 24 | 36 | 41 | 4 |
|  | 0.10 | 28 | 26 | 42 | 12 | 0 | 6 | 12 | 5 | 17 | 12 | 23 | 27 | 0 |
|  | 0.05 | 12 | 9 | 22 | 0 | 0 | 0 | 0 | 0 | 1 | 0 | 7 | 8 | 0 |
|  | 0.01 | 0 | 0 | 0 | 0 | 0 | 0 | 0 | 0 | 0 | 0 | 0 | 0 | 0 |
|  | 0.00 | 0 | 0 | 0 | 0 | 0 | 0 | 0 | 0 | 0 | 0 | 0 | 0 | 0 |

**Supplemental Table 4.** Descriptive statistics for chart completion efficiency, visit volume, and EHR use metrics with stratification by specialty as well as high and low chart completion efficiency and visit volume.

|  | Chart completion efficiency | | | Visit volume | | |
| --- | --- | --- | --- | --- | --- | --- |
| Specialty | % closed on same day (SD) | Difference in chart completion top-bottom quintile (%) | p-value | N/scheduled day (SD) | Difference in visit volume top-bottom quintile (%) | p-value |
| Cardiology | 72.21 (32.8) | 85.4 (85.7) | <0.001 | 9.5 (5.4) | 14.5 (82.0) | <0.001 |
| Dermatology | 77.23 (29.3) | 74.9 (74.9) | <0.001 | 16.55 (8.1) | 22.0 (77.6) | <0.001 |
| Endocrinology | 74.30 (29.9) | 77.8 (78.0) | <0.001 | 9.3 (4.6) | 12.3 (75.8) | <0.001 |
| Gastroenterology | 62.49 (34.7) | 91.8 (92.3) | <0.001 | 8.3 (4.2) | 11.0 (76.0) | <0.001 |
| Hematology | 52.19 (36.3) | 96.2 (98.1) | <0.001 | 8.3 (6.0) | 14.2 (84.1) | <0.001 |
| Nephrology | 70.60 (33.2) | 86.5 (86.5) | <0.001 | 7.6 (4.3) | 10.4 (76.5) | <0.001 |
| Neurology | 68.38 (32.8) | 86.5 (86.6) | <0.001 | 6.8 (3.7) | 9.7 (78.6) | <0.001 |
| OB-GYN | 80.83 (25.2) | 63.2 (63.4) | <0.001 | 12.9 (6.0) | 16.6 (77.2) | <0.001 |
| Oncology | 54.44 (37.2) | 97.5 (99.0) | <0.001 | 9.1 (5.3) | 14.0 (82.0) | <0.001 |
| Pediatrics | 75.69 (29.3) | 76.1 (76.3) | <0.001 | 12.4 (6.6) | 18.2 (83.7) | <0.001 |
| Primary Care | 76.64 (28.6) | 73.2 (73.5) | <0.001 | 12.4 (6.0) | 16.22 (77.5) | <0.001 |
| Psychiatry | 68.92 (34.8) | 90.1 (90.1) | <0.001 | 5.8 (3.9) | 10.0 (85.7) | <0.001 |
| Pulmonology | 72.80 (32.0) | 83.1 (83.1) | <0.001 | 7.6 (4.3) | 11.2 (78.9) | <0.001 |
|  |  |  |  |  |  |  |
| EHR use metrics | Min/scheduled day (SD) | Difference in chart completion top-bottom quintile (%) | p-value | Min/visit (SD) | Difference in chart completion top-bottom quintile (%) | p-value |
| Time on Inbox | 26.89 (22.7) | -1.8 (-7.1) | <0.001 | 3.3 (5.6) | -0.8 (-33.5) | <0.001 |
| Time on Documentation | 72.41 (49.2) | -4.9 (-7.0) | <0.001 | 8.9 (11.0) | -2.3 (-9.1) | <0.001 |
| Time on Orders | 28.55 (22.0) | 0.19 (0.7) | <0.001 | 3.0 (3.0) | -0.4 (-79.8) | <0.001 |
| Time on Visit Navigator | 12.3 (10.5) | 0.25(1.9) | <0.001 | 1.4 (1.7) | -0.2 (-29.7) | <0.001 |
| Time on Clinical Review | 34.5 (23.9) | -3.0 (-9.0) | <0.001 | 4.5 (6.5) | -1.1 (-4.3) | <0.001 |

**Supplemental Figure 2.** Confusion matrix for XGBoost multi-class classifier model predicting the proportion of charts completed.

**Supplemental Figure 3.** ROC Curves for each class for XGBoost multi-class classifier model predicting the proportion of charts completed.

**Supplemental Table 5.** Evaluation metrics of XGBoost multi-class classifier model performance in predicting proportion of charts completed.

|  | Top 20% (SD) | Second 20% (SD) | Middle 20% (SD) | Fourth 20% (SD) | Bottom 20% (SD) |
| --- | --- | --- | --- | --- | --- |
| Accuracy | 0.74 (<0.001) | 0.81(<0.001) | 0.76 (<0.001) | 0.74 (<0.001) | 0.83 (<0.001) |
| Precision (PPV) | 0.49 (0.001) | 0.33 (0.012) | 0.31(0.034) | 0.35 (0.007) | 0.56 (0.001) |
| Recall | 0.68 (0.001) | 0.16 (<0.001) | 0.21(<0.001) | 0.39 (<0.001) | 0.64 (0.001) |
| F1 | 0.57 (0.001) | 0.22 (<0.001) | 0.25 (<0.001) | 0.37 (<0.001) | 0.60 (0.001) |
| NPV | 0.87 (<0.001) | 0.85 (<0.001) | 0.83 (<0.001) | 0.85 (<0.001) | 0.91(<0.001) |
| AUC ROC | 0.81(<0.001) | 0.72 (<0.001) | 0.66 (<0.001) | 0.72 (<0.001) | 0.87 (<0.001) |

**Supplemental Figure 4.** SHAP summary plot for top 10 features in predicting high same day chart completion.

**Supplemental Figure 5.** XGBoost regression model actual vs. predicted value visit volume.

**Supplemental Figure 6.** ROC Curves for each class for XGBoost multi-class classifier model predicting visit volume per scheduled day.

**Supplemental Table 6.**  Evaluation metrics of XGBoost multi-class classifier model performance in predicting visit volume

|  | Top 20%  (SD) | Second 20%  (SD) | Middle 20%  (SD) | Fourth 20%  (SD) | Bottom 20%  (SD) |
| --- | --- | --- | --- | --- | --- |
| Accuracy | 0.84 (<0.001) | 0.76 (<0.001) | 0.77 (<0.001) | 0.78 (<0.001) | 0.89 (<0.001) |
| Precision (PPV) | 0.59 (0.001) | 0.41(0.004) | 0.40 (<0.001) | 0.45 (0.002) | 0.72 (0.001) |
| Recall | 0.70 (0.001) | 0.39 (<0.001) | 0.30 (<0.001) | 0.48 (<0.001) | 0.75 (0.001) |
| F1 | 0.64 (0.001) | 0.40 (<0.001) | 0.34 (<0.001) | 0.46 (0.001) | 0.74 (<0.001) |
| NPV | 0.92 (<0.001) | 0.85 (<0.001) | 0.84 (<0.001) | 0.87 (<0.001) | 0.94 (<0.001) |
| AUC ROC | 0.90 (<0.001) | 0.77 (<0.001) | 0.74 (<0.001) | 0.80 (<0.001) | 0.94 (<0.001) |

**Supplemental Figure 7.** SHAP summary plot for top 10 features in predicting visit volume per scheduled day.
